# Abscinazole-E3M, a practical inhibitor of abscisic acid 8′-hydroxylase for improving drought tolerance

**DOI:** 10.1038/srep37060

**Published:** 2016-11-14

**Authors:** Jun Takeuchi, Masanori Okamoto, Ryosuke Mega, Yuri Kanno, Toshiyuki Ohnishi, Mitsunori Seo, Yasushi Todoroki

**Affiliations:** 1 College of Global-Interdisciplinary Studies, Academic Institute, Shizuoka University, 836 Ohya, Suruga-ku, Shizuoka 422-8529, Japan; 2 Arid Land Research Center, Tottori University, 1390 Hamasaka, Tottori 680-0001, Japan; 3 PRESTO, Japan Science and Technology Agency, Kawaguchi, Saitama 332-0012, Japan; 4 RIKEN Center for Sustainable Resource Science, 1-7-22 Suehiro-cho Tsurumi-ku, Yokohama 230-0045, Japan; 5 College of Agriculture, Academic Institute, Shizuoka University, 836 Ohya, Suruga-ku, Shizuoka 422-8529, Japan; 6 Reseach Institute of Green Science and Technology, Shizuoka University, 836 Ohya, Suruga-ku, Shizuoka 422-8529, Japan; 7 Graduate School of Science and Technology, Shizuoka University, 836 Ohya, Suruga-ku, Shizuoka 422-8529, Japan

## Abstract

Abscisic acid (ABA) is an essential phytohormone that regulates plant water use and drought tolerance. However, agricultural applications of ABA have been limited because of its rapid inactivation in plants, which involves hydroxylation of ABA by ABA 8′-hydroxylase (CYP707A). We previously developed a selective inhibitor of CYP707A, (−)-Abz-E2B, by structurally modifying *S*-uniconazole, which functions as an inhibitor of CYP707A and as a gibberellin biosynthetic enzyme. However, its synthetic yield is too low for practical applications. Therefore, we designed novel CYP707A inhibitors, Abz-T compounds, that have simpler structures in which the 1,2,3-triazolyl ring of (−)-Abz-E2B has been replaced with a triple bond. They were successfully synthesised in shorter steps, resulting in greater yields than that of (−)-Abz-E2B. In the enzymatic assays, one of the Abz-T compounds, (−)-Abz-E3M, acted as a strong and selective inhibitor of CYP707A, similar to (−)-Abz-E2B. Analysis of the biological effects in *Arabidopsis* revealed that (−)-Abz-E3M enhanced ABA’s effects more than (−)-Abz-E2B in seed germination and in the expression of ABA-responsive genes. Treatment with (−)-Abz-E3M induced stomatal closure and improved drought tolerance in *Arabidopsis*. Furthermore, (−)-Abz-E3M also increased the ABA response in rice and maize. Thus, (−)-Abz-E3M is a more practical and effective inhibitor of CYP707A than (−)-Abz-E2B.

Water deficit is an adverse environmental stress that affects plant growth and agricultural productivity. To cope with drought, plants display a variety of physiological and biochemical responses at the cellular and whole-organism levels^1^. Various plant hormones, especially abscisic acid (ABA), regulate water consumption and drought tolerance in plants^2^,^3^. Under drought conditions, the endogenous ABA level dramatically increases in plants, which reduces transpiration rates by inducing stomatal closure^4^,^5^. Moreover, ABA induces an accumulation of compatible solutes, such as sorbitol, proline, glycine-betaine, and small hydrophilic proteins, that confer stress tolerance by decreasing the osmotic potential in plant cells^2^. Therefore, ABA can be considered a water stress-related phytohormone that contributes to the dehydration and desiccation tolerance of cells. Although ABA is registered as an agricultural chemical because it is a plant growth regulator, its practical use has been limited, mainly because of its weak effects in field trials^6^, which were caused by its rapid biodegradation.

ABA is inactivated by two major pathways: (i) hydroxylation of ABA’s C8′ position and (ii) glycosylation of ABA’s C1′ position. ABA-8′-hydroxlase is a P-450 type monooxygenase that is encoded by the CYP707A family, *CYP707A1–CYP707A4*, in the model plant *Arabidopsis thaliana*^7^,^8^, and many CYP707A isozymes have been found in various plant species^9^,^10^,^11^,^12^. The reaction product, 8′-hydroxy-ABA, is highly unstable; therefore, it spontaneously isomerises to phaseic acid (PA), which has a considerably lower hormonal activity than ABA^13^. However, ABA and its metabolites are also inactivated by conjugation to ABA-glucosyl ester (ABA-GE), which is stored in the vacuoles. This process is catalysed by glucosyltransferases, referred to as UDP-glucosyltransferases (UGTs), such as UGT71B6–UGT71B8^14^ and UGT71C5^15^. Previously, ABA-GE was considered a permanently inactivated form, but recent studies indicate that it is a storage or transport form of ABA. Under drought conditions, ABA-GE is cleaved by β-glucosidases that are present in the endoplasmic reticulum (AtBG1)^16^ and vacuoles (AtBG2)^17^, indicating that ABA glucosylation is a reversible process that is catalysed by glucosidases and hydro-lyases. Therefore, we have focused on ABA 8′-hydroxylase, which catalyses the irreversible pathway of ABA inactivation, to develop a specific inhibitor of ABA catabolism.

Compounds with a nitrogen-containing heterocycle, such as triazoles and imidazoles, are known to act as inhibitors of various P-450 enzyme-mediated hydroxylation events. Among them, *S*-uniconazole (UNI; Fig. 1), a plant growth retardant developed in the 1980s^18^, inhibits CYP707A^19^ and *ent*-kaurene oxidase (CYP701A), which catalyses the three-step oxidation of *ent*-kaurene to *ent*-kaurenoic acid (KA)^20^, a biosynthetic precursor of the plant hormone gibberellin (GA). Hence, treatment with UNI reduces water loss and enhances drought tolerance^19^; however, its low specific inhibition of P-450 enzymes limits its potential use as a chemical probe and in practical applications. To address this problem, we focused on the structure of UNI, which may be small and flexible enough to embed itself into the various substrate binding pockets of P-450 enzymes. This assumption led us to the possibility that enlarging or conformationally restricting the UNI molecule could result in a narrow inhibition spectrum. Accordingly, in earlier work^21^, we modified the structure of UNI to develop a specific inhibitor of CYP707A using three approaches: (i) molecular enlargement^22^; (ii) conformational restriction^23^; and (iii) azole ring modification^24^. Of these, the molecular enlargement approach was the most effective and enabled us to develop specific inhibitors of CYP707A, UT4^22^, abscinazole-E1^25^, and abscinazole-E2B (Abz-E2B)^26^. Abz-E2B inhibited CYP707A3 effectively *in vitro* and enhanced drought tolerance in plants through a temporary increase in the endogenous ABA level without growth inhibition. These results indicated that Abz-E2B is promising, not only as a chemical probe for dissecting the various roles of ABA but also as a plant growth regulator.

Despite the potential value of Abz-E2B for imparting drought-stress resistance to plants, the synthetic yield of 1% is too low for practical uses in agriculture and horticulture. Therefore, in the present study, we designed novel CYP707A inhibitors, abscinazole-T (Abz-T) compounds (**3**–**6**; Fig. 1), which have simpler structures, with a triple bond instead of the 1,2,3-triazolyl ring of Abz-E2B, and their syntheses resulted in greater yields (15–28% overall yield) compared with that of Abz-E2B (1% overall yield). Here, we describe the design and preparation of the Abz-T compounds, and their effects on CYP707A and CYP701A both *in vitro* and *in vivo*. Of the eight initial inhibitors, including their respective enantiomers, (−)-compound **6**, referred to as (−)-Abz-E3M, enhanced ABA’s effects more than (−)-Abz-E2B, and treatments of (−)-Abz-E3M led to stomatal closure and enhanced ABA responses in both *Arabidopsis* and maize. Thus, (−)-Abz-E3M is a more practical inhibitor of CYP707A than (−)-Abz-E2B when used as a plant growth regulator.

## Results

### Design and synthesis of novel CYP707A inhibitors: Abz-T compounds

**Figure 1:**
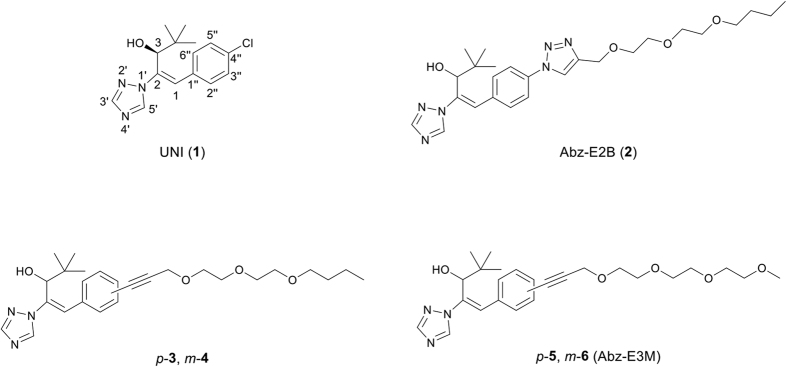
Structures of UNI, Abz-E2B and novel CYP707A inhibitors.

The 1,2,4-triazolyl ring and the long ethylene glycol chain of Abz-E2B are critical for the effective inhibition of CYP707A. To function as a specific inhibitor of CYP707A, previous studies indicated that an enlarged analogue of UNI requires a longer alkyl chain to increase the selectivity for CYP707A over CYP701A^22^. However, because the longer alkyl chain results in greater hydrophobicity, it was replaced by the ethylene glycol chain^25^,^26^. It remains unclear whether the 1,2,3-triazolyl ring, which enters the *p*-position of the phenyl group in UNI by a click reaction^27^, of Abz-E2B is required for the selective inhibition of CYP707A. Additionally, the click chemistry requires three steps (amination, azidation and click reaction), which was the leading cause of the low synthetic yield of Abz-E2B. Thus, we designed the Abz-T compound series (compounds **3**–**6**), in which the 1,2,3-triazolyl ring of Abz-E2B was replaced with a triple bond to increase the synthetic yield (Fig. 1). Because of the superposition of optimised compound **3**, which has the triple bond at the *p*-position of the phenyl group, the optimised Abz-E2B resulted in the introduced side chains being oriented in different directions (Fig. S1). Compound **4**, with a side chain in the *m*-position was also designed. The introduction of the triple bond instead of the triazolyl ring may decrease water solubility because the calculated log *P* values [octanol–water partition coefficients (calculated value)] of compounds **3** and **4** are greater than that of Abz-E2B (calculated log *P* = 3.42). Therefore, we also designed compounds **5** and **6** (calculated log *P* = 3.00), which have methoxyethyl groups instead of the butyl groups of compounds **3** and **4**. This should decrease the log *P* value to less than 4.00, increasing the water solubility^22^.

**Figure 2:**
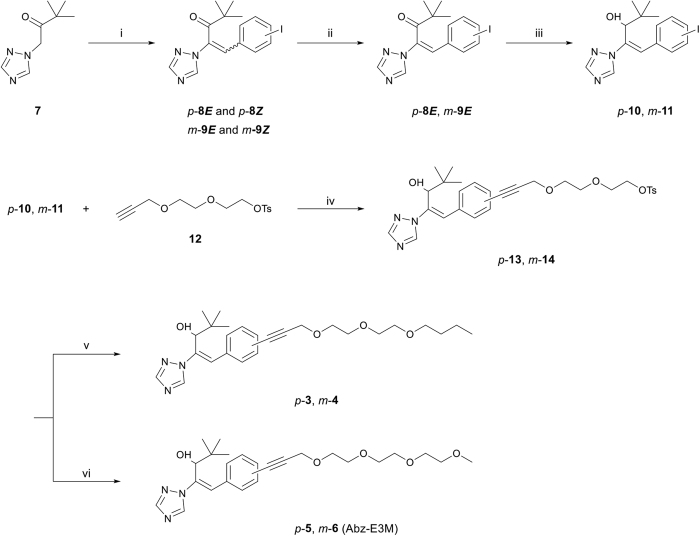
Synthesis of compounds 3–6. Reagents and conditions: (i) 4- or 3-iodobenzaldehyde, K_2_CO_3_, Ac_2_O, 100 °C; (ii) UV 365 nm, MeOH, room temperature (RT); (iii) NaBH_4_, MeOH, RT; (iv) Pd(PPh_3_)_2_Cl_2_, Cul, Et_3_N, THF, RT; (v) NaH, 1-butanol, RT; (vi) NaH, 2-methoxyethanol, 60–80 °C.

Racemic Abz-T compounds were synthesised from 3,3-dimethyl-1-(1H-1,2,4-triazol-1-yl)butan-2-one (**7**) as shown in Fig. 2. The aldol condensation of **compound 6** and 4-iodobenzaldehyde, or 3-iodobenzaldehyde, resulted in *E*/*Z* mixtures of ketones **8** and **9** (2:9 for ketone **8** and 3:10 for ketone **9**), which were UV-irradiated (365 nm) to increase the population of the *E*-isomers by photoisomerization. These *E*-ketones were reduced to yield **10** and **11**. The side chain (**12**) was introduced by Sonogashira cross-coupling using copper (I) and *trans*-dichlorobis(triphenylphosphine)Pd (II), generating compounds **13** and **14**, which were treated with sodium hydride in 1-butanol or 2-methoxyethanol to produce (±)-Abz-T compounds (**3**–**6**). These four compounds were successfully synthesised as racemic mixtures in shorter steps, with better yields (six steps, 15–28% overall yield) compared with those of Abz-E2B (eight steps, 1% overall yield). All of the compounds were optically resolved by high-performance liquid chromatography (HPLC) on a chiral column before testing their effects *in vitro* and *in vivo*.

### Enzymatic selectivity of Abz-T compounds

The inhibitory activities of Abz-T compounds against ABA 8′-hydroxylase were examined using recombinant *Arabidopsis* CYP707A3 co-expressed with *Arabidopsis* P-450 reductase (ATR1) in *Escherichia coli*^22^. The activity levels were evaluated based on the decrease in the amount of the enzymatic product, PA. All of the (−)-Abz-T compounds showed a stronger inhibitory activity than the (+)-isomers, and the compounds with a side chain at the *m*-position of the phenyl group (compounds **4** and **6**) inhibited CYP707A3 more strongly than the compounds with a side chain at the *p*-position (compounds **3** and **5**; Table 1). The concentration of inhibitor necessary to halve the response (IC_50_) of (−)-compound **4** and (−)-compound **6** were comparable with that of (−)-Abz-E2B when 5 μM ABA was used as the substrate. The inhibition constant (*K*_I_) of (−)-compound **6** was determined to be 35 nM. Thus, the affinity of (−)-compound **6** for the CYP707A3 active site may be nearly equivalent to that of (−)-Abz-E2B (*K*_I_ = 38 nM), and hence, the 1,2,3-triazolyl ring of Abz-E2B does not control the affinity for the CYP707A active site.

To evaluate the enzymatic selectivity between CYP707A and CYP701A, we examined the inhibitory activity against recombinant rice containing CYP701A6 expressed in insect cells using a baculovirus vector. This enzyme catalyses all three steps to KA via the corresponding alcohol and aldehyde^20^, and consequently, the kinetic analysis is very complicated. Therefore, we evaluate the IC_50_ value for the formation of the end product, KA, instead of the *K*_I_ value. The IC_50_ values of all of the compounds, except for compound **4**, were at least 10-fold higher than that of UNI (IC_50_ = 57 nM) (Table 1), which was similar to the results previously reported for (−)-Abz-E2B, indicating that the inhibitory activity against CYP701A was substantially reduced compared with that of UNI. This result, together with the strong effect on CYP707A, suggested that the replacement of the 1,2,3-triazolyl ring with a triple bond had little effect on the enzymatic selectivity between CYP707A and CYP701A, and we determined that (−)-compound **6** was the best candidate selective inhibitor of CYP707A *in vitro*.

### Physiological effects of Abz-T compounds on plants

**Figure 3:**
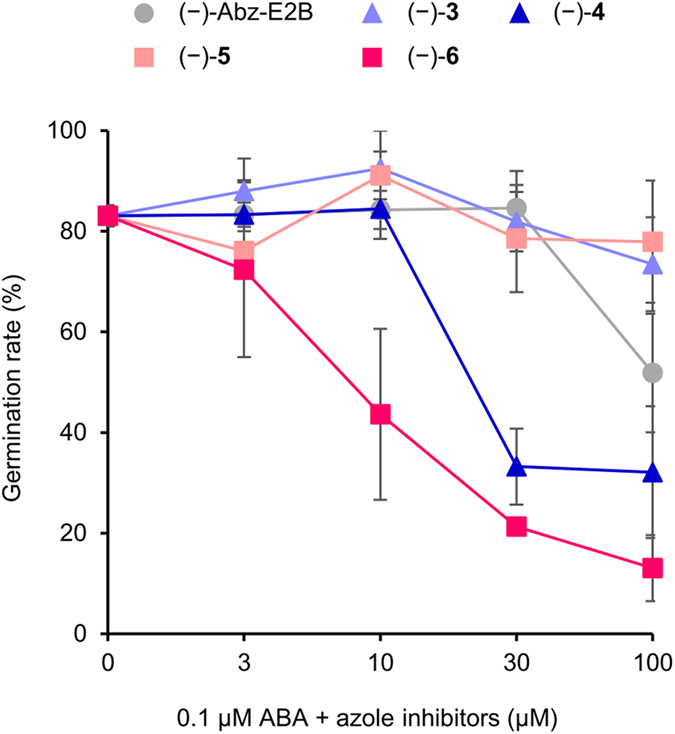
Effects of (−)-Abz-T compounds on *Arabidopsis* seed germination compared with that of (−)-Abz-E2B. Seed germination rates in the presence of 0.1 μM ABA and the indicated concentrations of azole inhibitors at a time when the germination rate of plants treated only with ABA was 80% (*n* = 3; error bars represent s.d.). Compound **6** is also referred to as Abz-E3M.

The effects of (−)-Abz-T compounds, which are more active enantiomers, were examined first using the *Arabidopsis* seed germination assay, which relies on ABA’s inhibitory effect on germination to evaluate the inhibitory activity against CYP707A in plants. In the absence of ABA, each compound showed no effect at a concentration range of 3 to 100 μM (Fig. S2). Because the most commonly used *Arabidopsis* ecotype Col-0 does not undergo deep seed dormancy, the effects of ABA catabolic inhibitors may be difficult to determine under standard laboratory growth conditions in which the endogenous ABA content is kept at a very low level. However, in the presence of ABA, compounds (−)-**4** and (−)-**6** enhanced the effects of ABA, and the potency of (−)-compound **6** was 10-fold greater than that of (−)-Abz-E2B (Fig. 3), although it showed almost the same *K*_I_ value as (−)-Abz-E2B in the *in vitro* CYP707A3 inhibition assay. In addition, (−)-compound **6** inhibited *Arabidopsis* seed germination under high temperature conditions (Fig. S3), which can be mimicked in the laboratory to cause thermoinhibition and induce the accumulation of endogenous ABA in plants^28^.

**Figure 4:**
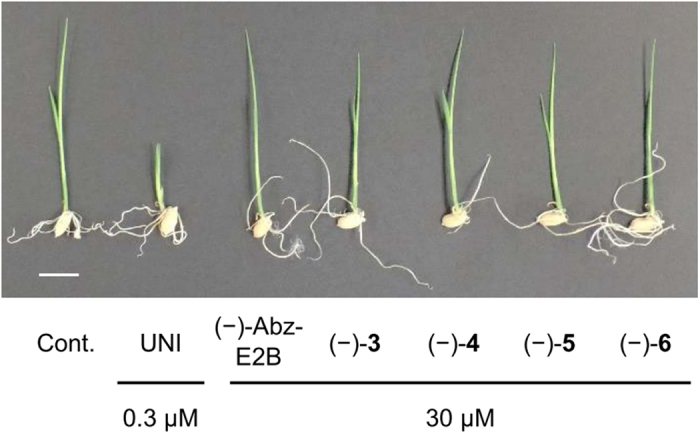
Effects of (−)-Abz-T compounds on rice seedling growth compared with the effects of UNI and (−)-Abz-E2B. Seedlings grown on test media containing indicated concentrations of azole inhibitors for 7 d. Scale bars represent 10 mm. Compound **6** is also referred to as Abz-E3M.

The effects of (−)-Abz-T compounds were also tested on the early growth of rice seedlings to investigate whether the plant growth-retardant effect derived from UNI declined. UNI strongly inhibited the elongation of rice seedlings, even at 0.3 μM, under our assay conditions. In the same bioassay, none of the four (−)-Abz-T compounds affected seedling growth when present in a 100-fold excess over UNI (Fig. 4), although they weakly inhibited seedling growth when present at a high 100 μM concentration (Fig. S4). This was the same trend observed for (−)-Abz-E2B^26^. Considering that even (−)-compound **4**, which inhibited CYP701A6 with the same potency as UNI, did not inhibit seedling growth at less than 100 μM, the growth-retardant effects of Abz-T compounds may not be caused by the inhibition of CYP701A. In addition, the retardant effect of (−)-compound **6** was additively enhanced by ABA (Fig. S5). That is, a co-treatment of 10 μM (−)-compound **6** and 1 μM ABA inhibited rice seedling growth but treatments of each compound independently did not. Thus, the growth-retardant effects of (−)-Abz-T compounds may arise from an increase in the endogenous ABA level due to the CYP707A inhibition, although we cannot exclude the possibility that Abz-T compounds may inhibit off-target enzymes other than CYP707A and CYP701A.

Based on the above-mentioned enzymatic and physiological assays, (−)-compound **6** is the most ideal CYP707A inhibitor among the tested compounds. We named compound **6** as abscinazole-E3M (Abz-E3M) before testing it in additional physiological and biological assays. The absolute stereochemistry of both enantiomers of Abz-E3M was determined by the advanced Mosher’s method^29^ using the ^1^H nuclear magnetic resonance spectra of their *R*-α-methoxy-α-(trifluoromethyl)phenyl acetates (MTPA). The *R*-MTPA esters were prepared with *S*-MTPA-Cl in pyridine. The vinyltriazole protons of the (−)-isomer and the *t*-butyl protons of the (+)-isomer moved to a higher magnetic field (high field shift) owing to the magnetic anisotropy effect of the phenyl group in MTPA. Therefore, the absolute configurations of the (−)- and (+)-isomers were determined to be *S* and *R*, respectively. Based on the determination, *S*-(−)-Abz-E3M should be a more effective inhibitor of CYP707A. This is inconsistent with the case of Abz-E2B, which had an *R*-isomer that was more potent than its *S*-isomer^26^. These observations suggested that the differences in the stereostructures around the chiral carbons of Abz-E3M and Abz-E2B did not strictly affect the recognition by CYP707A.

### Comparisons of the effects of (−)-Abz-E3M and (−)-Abz-E2B on the expression levels of ABA-responsive genes

**Figure 5:**
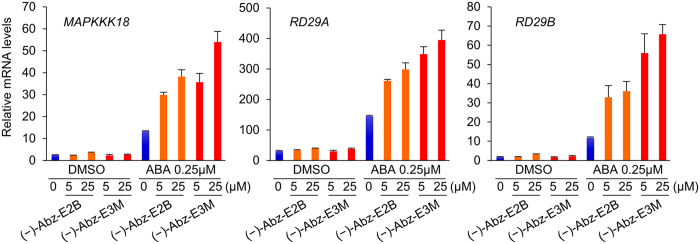
Effects of (−)-Abz-E3M on the expression levels of Arabidopsis ABA-responsive genes. Expression of *MAPKKK18, RD29A* and *RD29B* after treatment with (−)-Abz-E2B or (−)-Abz-E3M in the absence or presence of 0.25 μM ABA (*n* = 4, error bar represents s.d.). Ten-day-old seedlings of wild-type (Col accession) were incubated in solutions containing the chemicals for 6 h.

The molecular bases of (−)-Abz-E3M actions *in vivo* was further characterised in *Arabidopsis*. In the absence of exogenous ABA, (−)-Abz-E3M and (−)-Abz-E2B did not induce the expression of the ABA-responsive genes *MAPKKK18, RD29A* and *RD29B* (Fig. 5). However, as co-treatments with ABA, both compounds enhanced the expression levels of ABA-responsive genes, and the potency of (−)-Abz-E3M was greater than that of (−)-Abz-E2B, which was consistent with the *Arabidopsis* seed germination assay. Moreover, tissue-specific ABA responses were monitored after (−)-Abz-E3M treatment using an *Arabidopsis* transgenic reporter line in which the β-glucuronidase (GUS) gene was introduced under control of the *MAPKKK18* promoter. This promoter generally expresses in the vascular tissues of both leaves and roots in response to ABA. Consistent with the quantitative reverse transcription-polymerase chain reaction (qRT-PCR) analysis, (−)-Abz-E3M enhanced the ABA-inducible GUS staining when used as a co-treatment with ABA (Fig. S6), because (−)-Abz-E3M inhibited ABA catabolism and increased ABA accumulation in plants (Fig. S7).

### The effects of (−)-Abz-E3M on stomatal closure and drought tolerance

**Figure 6:**
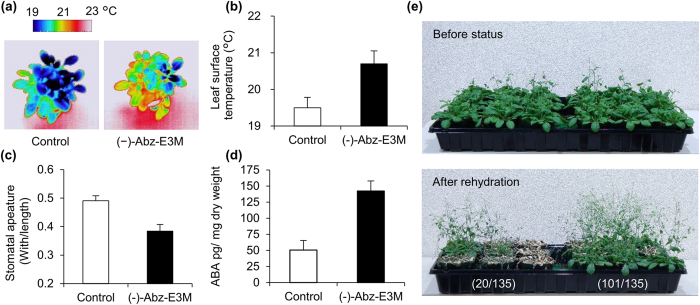
Effects of (−)-Abz-E3M on *Arabidopsis* drought tolerance. (**a**,**b**) Three-week-old plants were treated with 50 μM of (−)-Abz-E3M and incubated overnight at 22 °C with 60% relative humidity. Thermal images of plants were obtained using a thermal imaging camera (TH9100WR, Nippon Avionics Co., Ltd.), and the average temperatures on leaf surfaces were determined with NS9200 software (*n* = 5, error bars represent s.d.). Representative images are shown in (**a**). (**c**) Stomatal apertures after the application of 50 μM (−)-Abz-E3M. After an overnight incubation, plants were placed at 22 °C with ~90% relative humidity for 1 h. Stomatal images were obtained using Suzuki’s universal micro-printing method. Values are means ± SEM (*n* = 5, ~30 stomata per individual leaf). (**d**) Effects of (−)-Abz-E3M on endogenous ABA levels. Four-week old plants grown in soil pots were treated with 50 μM (−)-Abz-E3M, and leaves were harvested after an overnight incubation. Values are means with s.d. (*n* = 6). (**e**) Four-week-old plants were subjected to drought stress by withholding water. Initially, 50 mL of 50 μM (−)-Abz-E3M containing 0.05 % Tween-20 (or a no chemical control treatment) was sprayed three times every other day on 135 *Arabidopsis* plants. Plants were rehydrated after a 12-d drought treatment, and their subsequent recovery statuses were photographed after 3 d. The number of surviving plants per total number of tested plants is inset in the photograph.

**Figure 7:**
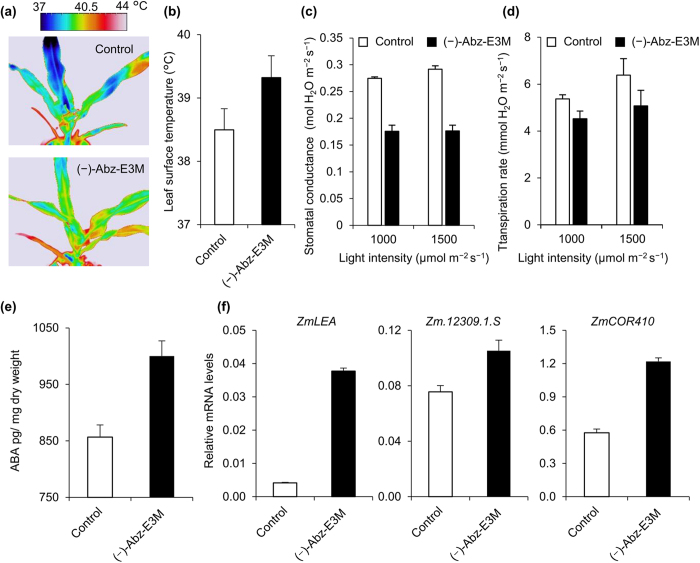
Effects of (−)-Abz-E3M on maize. (**a**) Thermal images of the leaf surface temperatures of (−)-Abz-E3M-treated maize plants. (**b**) Leaf surface temperatures of (−)-Abz-E3M-treated maize plants. The stomatal conductance (**c**) and transpiration rate (**d**) in (−)-Abz-E3M-treated maize at different light intensities. (**a**–**d**) One-month-old plants were used in this experiment, and 10 mL of 100 μM (−)-Abz-E3M was sprayed per plant, with data obtained at 30,000 ± 1,500 lux, 41.5 ± 1.5 °C and 35 ± 5% relative humidity after an overnight incubation. Values are means with s.d. (*n* = 4 for leaf surface temperature, *n* = 3 for stomatal conductance and transpiration rate). (**e**) Effects of (−)-Abz-E3M on endogenous ABA levels. (**f**) Expression levels of *ZmLEA, Zm.12309.1.S* and *ZmCOR410* after the (−)-Abz-E3M treatment. (**e**,**f**) Root parts of 2-week-old whole seedlings were dipped in 100 μM (−)-Abz-E3M chemical solution containing 0.05% Tween-20 overnight, and aerial shoot tissues were used for qRT-PCR and ABA measurement analyses. Values are means with s.d. (*n* = 3 for qRT-PCR, *n* = 5 for ABA measurement).

The effects of (−)-Abz-E3M were also determined relative to stomatal apertures. *Arabidopsis* grown in soil pots underwent moderate drought stress under the optimal growth conditions of 22 °C and 60% relative humidity. Under these growth conditions, the leaf surface temperature of the mock-treated controls was 19.5 °C, which was lower than the surrounding atmospheric temperature because of transpirational water loss (Fig. 6a,b)^30^. In contrast, treatments with (−)-Abz-E3M reduced the water loss and showed increased leaf temperatures (Fig. 6a,b). Consistent with the leaf surface temperatures, treatments with (−)-Abz-E3M led to stomatal closure (Fig. 6c). In addition, the effects of (−)-Abz-E3M on endogenous ABA levels were also investigated, and (−)-Abz-E3M was found to significantly increase the ABA content in *Arabidopsis* (Fig. 6d). Finally, we examined the effects of (−)-Abz-E3M on drought tolerance under more practical conditions. Three-week-old *Arabidopsis* were sprayed with an aqueous solution containing 50 μM (−)-Abz-E3M. Water was not supplied for 12 days, and then the plants were rehydrated. The application of (−)-Abz-E3M before withholding water induced significant drought tolerance and increased the survival rate (Fig. 6e). We also tested the effects of (−)-Abz-E3M on a monocot crop, maize, to evaluate whether (−)-Abz-E3M affected stomatal movement and enhanced ABA response across plant species. As was the case in *Arabidopsis*, treatment with (−)-Abz-E3M increased the leaf surface temperature, and suppressed stomatal conductance and transpiration rates compared with the mock-treated control (Fig. 7a–d). Moreover, (−)-Abz-E3M increased the endogenous ABA levels (Fig. 7e) and consequently induced the expression of ABA-responsive genes, such as *ZmLEA, Zm.12309.1.S* and *ZmCOR410* (Fig. 7f). Thus, the effects of (−)-Abz-E3M were not restricted to the model plant *Arabidopsis*.

### The facile preparation of optically active (−)-Abz-E3M

In the above-mentioned enzymatic, physiological and biological assays, we used (−)-Abz-E3M that was optically resolved by HPLC on a chiral column. This method enabled us to isolate the optically active (−)-enantiomer in high excess (>99% ee), but it was time consuming and difficult. To be able to put (−)-Abz-E3M into practical use in the future, we devised a facile method to separate (−)-Abz-E3M from the racemate. Racemic Abz-E3M was first converted to a diastereomeric mixture using a chiral derivatising agent, *N*-(*p*-toluenesulfonyl)-L-phenylalanyl chloride. In this reaction, the (+)-enantiomer preferentially reacted with the reagent, allowing us to successfully isolate unreacted (−)-Abz-E3M in high enantiomeric excess (Fig. S8). When using a reagent of the five equivalents, based on racemic Abz-E3M, its (−)-enantiomer was obtained in 94% ee. Because (–)-Abz-E3M showed no significant adverse effects in enzymatic and physiological assays, the (−)-Abz-E3M prepared by this separation method can be used both as a chemical tool and as a plant growth regulator.

## Discussion

We designed four Abz-T compounds, structural analogues of the previously developed ABA 8′-hydroxylase (CYP707A) inhibitor, Abz-E2B, to increase the practical use of such an inhibitor by improving the synthetic yield. These compounds were successfully synthesised in shorter steps and had better yields (six steps, 15–28% overall yield) compared with those of Abz-E2B. In the enzymatic, physiological and biological assays, these new compounds, especially (−)-Abz-E3M, showed similar overall ligand profiles to that of (−)-Abz-E2B. Thus, they had strong inhibitory activities against CYP707A, and the weak inhibitory activities against CYP701A. Interestingly, (−)-Abz-E3M enhanced ABA’s effects by 10-fold compared with (−)-Abz-E2B in the *Arabidopsis* seed germination assay as a co-treatment with ABA, although both compounds showed almost the same *K*_I_ values against recombinant CYP707A3. This suggested that the inhibitory profiles from *in vitro* enzyme assays using the recombinant enzymes may not fully correspond to *in vivo* enzyme reactions. Because plant phenotypes can be affected by many factors, such as uptake, metabolism and unexpected side effects, other than the direct action of the target enzyme, it is difficult to explain why (−)-Abz-E3M acted as a more potent inhibitor of CYP707A than (−)-Abz-E2B in plants. However, we considered several possibilities. For example, (−)-Abz-E3M may have a longer residence time in the CYP707A active site compared with (−)-Abz-E2B, because the biological effects produced by a ligand-enzyme complex are directly related to the binary complexes’ lifetime. The longer the ligand is in residence at its target, the longer the biological effect lasts^31^. Thus, the dissociation rate constant value of (−)-Abz-E3M for CYP707A may be less than that of (−)-Abz-E2B.

Conversely, in the case of Abz-E2B, *S*-(−)-Abz-E3M showed a stronger inhibitory activity than its *R*-(+)-enantiomer. Based on the inhibition patterns of azole compounds against P-450 enzymes, the 1,2,4-triazolyl rings of both *S*-(−)-Abz-E3M and *R*-(−)-Abz-E2B coordinate with the heme iron in the active site of CYP707A. In addition, because both compounds inhibited CYP707A with potencies at levels comparable to that of UNI, their long side chains may extend to the spatially wide entrance of the CYP707A ligand-binding pocket, although this cannot be discussed in greater detail owing to CYP707A’s lack of structural characterization. The largest structural differences between *S*-(−)-Abz-E3M and *R*-(−)-Abz-E2B in the ligand-binding pocket of CYP707A should be the directions of their *t*-butyl and hydroxy groups, and these substituents in both compounds may not be important for binding to CYP707A. Such an inversion, accompanying the structural modifications of chiral compounds, has also been observed between Abz-E2B and its parent compound, UNI^26^.

The ABA catabolic enzyme has been an attractive target for regulating the endogenous ABA level in plants, and thus, its inhibitor, (−)-Abz-E2B, is expected to be useful as a plant growth regulator, in addition to acting as a research tool for chemical biology and chemical genetics. However, its practical use has been limited mainly owing to a low synthetic yield. The present creation and characterization of a novel CYP707A inhibitor, (−)-Abz-E3M, now allows for the practical application of ABA’s effects in agricultural and horticultural fields. (−)-Abz-E3M, for example, can enhance the ABA response, not only in *Arabidopsis* but also in maize. Although these effects were not surprising, they highlight the potential use of (−)-Abz-E3M as a practical application to improve drought tolerance in plants.

## Methods

### CYP707A3 inhibition assay

Reaction mixtures, containing 25 μg mL^−1^ of CYP707A3 microsomes (co-expressed with ATR1 in *E. coli*), ABA (final concentrations of 1–64 μM for determining the *K*_I_ value or 5 μM for determining the IC_50_ value), inhibitors (0 for control, 0.5–1,500 μM in 5 μL dimethylformamide) and 130 μM NADPH in 100 mM potassium phosphate buffer (pH 7.25), were incubated at 30 °C for 10 min. Reactions were initiated by adding NADPH, stopped by the addition of 25 μL of 1 M NaOH and then acidified with 50 μL of 1 M HCl. Reaction products were extracted by loading the mixture onto an Oasis HLB cartridge (1 mL, 30 mg; Waters Corp., Milford, MA, USA) and were washed with 1 mL of 10% MeOH in H_2_O (v/v) containing 1% acetic acid (AcOH, v/v). The enzyme products were then eluted with 1 mL of MeOH containing 1% AcOH, and the eluate was concentrated *in vacuo*. Each dried sample was then dissolved in 50 μL of MeOH, and a 20-μL volume was subjected to HPLC (Prominence; Shimadzu Corp., Kyoto, Japan). HPLC conditions were as follows: the octadecylsilyl column was composed of Hydrosphere C18 (150 × 6.0 mm, YMC Co., Ltd., Kyoto, Japan), and the solvent comprised 17% acetonitrile (MeCN) in H_2_O containing 0.05% AcOH (v/v). The flow rate was 1.0 mL min^−1^, and detection was at 254 nm. Enzyme activity was evaluated by determining the amount of PA in control experiments prior to taking each set of measurements. The inhibition constants were determined using the Enzyme Kinetics module of the SigmaPlot 10 software (Systat Software, Inc., San Jose, CA, USA) after determining the inhibition mode by plotting the reaction velocity in the presence and absence of the inhibitor on a double-reciprocal plot. The inhibition ratio was defined as [(*A* − *B*)/*A*] × 100, where *A* = the amount of PA when the inhibitor was absent from the reaction mixture (control), and *B* = the amount of PA when the inhibitor was present. All of the tests were conducted at least three times.

### CYP701A6 inhibition assay

Reaction mixtures, containing 0.058 μM of CYP707A3 microsomes, ATR2 (0.115 U), *ent*-kaurene (final concentration of 10 μM), inhibitors (0 for control, 1.5–1,500 μM in 8 μL dimethylformamide), and 20 mM NADPH in 500 mM potassium phosphate buffer (pH 7.4), were incubated at 30 °C for 10 min. Reactions were initiated by adding NADPH and stopped by the addition of 100 μL of 1 M HCl. Abietic acid, as the internal standard, was then added to the reaction product, which was extracted with EtOAc (400 μL × 3). The organic phase was dried over Na_2_SO_4_ and concentrated *in vacuo*. To derivatize the reaction products, MeOH (50 μL) and trimethylsilyldiazomethane (2 M in 5 μL hexane) were added, and the reaction mixture was incubated at room temperature for 10 min. The organic solvent was removed under N_2_, and samples were adjusted to 150 μL with hexane before a gas chromatography-mass spectrometry analysis (QP2010-plus; Shimadzu Corp., Kyoto, Japan). The conditions were as follows: the column was DB-5ms (0.25 mm id × 15 m, 0.25 μm film thickness; Agilent J & W, Folsom, CA, USA), the carrier gas was He, the flow rate was 1.65 mL min^−1^, the injection port temperature was 280 °C, splitless injection, and the programmed column temperature was a step gradient of 80 °C for 1 min, 80–200 °C at 18 °C min^−1^, 200–210 °C at 2 °C min^−1^, 210–280 °C at 30 °C min^−1^, and then 280 °C for 3 min. The KA content was calculated from the area ratio of molecular and fragment ions of methyl KA (*m/z* 316, 273, and 257) to those of methyl abietate (*m/z* 316 and 256). The inhibition ratio was defined as [(*A* − *B*)/*A*] × 100, where *A* = the amount of methyl KA/methyl abietate when the inhibitor was absent from the reaction mixture (control), and *B* = the amount of methyl KA/methyl abietate when the inhibitor was present. All of the tests were conducted at least three times.

### Quantitative RT-PCR Analysis

*Arabidopsis* (Columbia accession) were grown for 10 d on agar plates containing 1/2 Murashige and Skoog (MS) and 0.5% sucrose at 22 °C under an 18/6-h light/dark cycle. Seedlings were incubated in the chemical solutions for 6 h. Total RNA from seedlings was isolated using the Plant RNA purification reagent (Thermo Fisher Scientific) according to the manufacturer’s instructions. cDNA was synthesised using the QuantiTec reverse transcription kit (Qiagen). Maize (cv. Gold rush, Sakata Seed Corporation) seedlings were grown in 100-mL pots containing 75 g of moist soil in a glass house. The root parts of the maize seedlings were washed with tap water and dipped in 100 μM (−)-Abz-E3M chemical solution containing 0.05% Tween-20 overnight. Total RNA from maize seedlings was isolated using an RNeasy mini kit (Qiagen) with the ‘on-column’ DNA digestion method. cDNA was synthesised using a High-Capacity cDNA Reverse Transcription Kit (Thermo Fisher Scientific). Real-time PCR using SYBR *Premix Ex Taq*™ II (Takara) was performed with Step One-Plus (Thermo Fisher Scientific). The primer sets are shown in Supplemental Table S1.

### ABA-responsive reporter analysis

MAPKKK18::GUS reporter-containing transgenic *Arabidopsis*^32^ were grown for 6 d on agar plates containing 1/2 MS and 0.5% sucrose. Seedlings were incubated in 1/2 MS solutions containing 0.5% sucrose with chemicals for 6 h. GUS activities were detected using a reaction buffer containing 50 mM sodium phosphate buffer, 2.5 mM potassium ferrocyanide, 2.5 mM potassium ferricyanide and 1 mM X-gluc. The GUS enzyme reaction was performed at 30 °C for ~12 h. The reaction was stopped by adding 70% EtOH. Tissue-specific GUS staining was observed by microscope after bleaching the chlorophyll pigment.

### Physiological assays for *Arabidopsis*, rice and maize

For the *Arabidopsis* seed germination assay, 25 to 40 seeds (Columbia accession) were sterilised successively by soaking in 70% EtOH for 30 min and reagent-grade EtOH for 1 min. They were then soaked in distilled water and incubated in the darkness at 5 °C for 3 d. The stratified seeds were then soaked in 100 μL of a test medium liquid agar in 96-well plates and allowed to germinate under continuous illumination at 22 or 30 °C. In these assays, we used the classic definition of radical emergence for seed germination. All of the assays were conducted at least three times.

For the rice seedling elongation assay, seven seeds (*Oryza sativa* L. cv. Nipponbare) were sterilised with reagent-grade EtOH for 5 min and washed with running tap water. They were then soaked in distilled water and incubated under continuous illumination at 25 °C for 3 d to germinate. The germinated seeds were then soaked in 2 mL of a test medium in a glass tube and grown under continuous illumination at 25 °C. When the seedlings were 7 d old, the length of the second leaf sheath was measured. All of the assays were conducted at least three times.

For the thermal imaging analysis in *Arabidopsis*, plants were grown for three weeks at 22 °C under an 18/6-h light/dark cycle. Four plants were grown per 100-mL pot, which contained 75 g of moist soil. Then, 25 mL of 50 μM (−)-Abz-E3M containing 0.05% Tween-20 was sprayed on 16 pots. After an overnight incubation, the leaf surface temperatures were monitored using a thermal imaging camera (TH9100WR, Nippon Avionics Co., Ltd.) at ~22 °C with ~60% relative humidity under light conditions. Temperatures on the leaf surfaces were analysed by NS9200 software (Nippon Avionics Co., Ltd.), and the average temperature difference between the control and (−)-Abz-E3M treatments was determined from 16 plants. To measure the stomatal apertures in *Arabidopsis*, stomatal images were obtained by Suzuki’s universal micro-printing method as described previously^32^. The copied stomatal images were photographed under microscopy, and then stomatal apertures were determined from the pore widths using ImageJ 1.50i softwater^33^ (National Institutes of Health).

For the drought tolerance analysis, five 2-week-old *Arabidopsis* seedlings were transferred into 200-mL pots containing 30 g of a 1:1 dry mixture of vermiculite:Promix with 120 g water, and the plants were grown for an additional two weeks at ~22 °C, with ~60% relative humidity under an 18/6-h light/dark cycle. After adjusting, by watering, to 150 g of moist soil (containing the five plants), 50 mL of 50 μM (−)-Abz-E3M containing 0.05% Tween-20 was sprayed on 27 pots, with a total 135 plants. Drought stress was started by withholding water. For the control treatment, 50 mL of water containing 0.05% Tween-20 and 100 μL MeOH, which was the same amount of carrier organic solvent in the chemical treatment, was sprayed on the same number of plants as the (−)-Abz-E3M treatment. The chemical solution was sprayed three times every other day after starting the drought-stress treatment. Plants were rehydrated after a 12-d drought treatment, and, subsequently, the recovery statuses were monitored to determine plant survival.

For thermal imaging and the transpirational water loss analysis in maize, plants were grown in 1/5,000-a Wagner pots containing 1 kg of dry horticultural soil (CAINZ Corporation) with 500 g of water, and the pots were maintained outside from July through August in Tottori City, Japan. Then, 10 mL of 100 μM (−)-Abz-E3M was sprayed on the 4-week-old plants, and the plants were relocated into a glass house. After an overnight incubation, the conductance and transpiration rate were analysed using an LI-6400 (Li-Cor Biosciences), and the leaf surface temperatures were measured using a thermal imaging camera (TH9100WR, Nippon Avionics Co., Ltd.) in the glass house. Temperatures on the leaf surfaces were analysed by NS9200 software (Nippon Avionics Co., Ltd.).

### Measurement of endogenous ABA levels

The extraction and purification of ABA was performed as described previously^34^ with some modifications. For ABA measurements from maize leaves, samples (150–400 mg dry weight) were ground with beads in 50-mL tubes and homogenised in 30 mL 80% (v/v) MeCN containing 1% AcOH (v/v) with *d*_6_-ABA (Icon Isotopes) as an internal standard. After extracting for 16 h at 4 °C, samples were centrifuged at 4 °C for 10 min at 3,000× *g*, and supernatants were collected. Samples were re-extracted twice with 20 mL 80% MeCN at 4 °C for 30 min, and the supernatants were collected after centrifugation. MeCN was removed using a nitrogen gas, and the acidified water extracts were loaded into 3cc Oasis HLB cartridges (Waters). After washing with water containing 1% AcOH, ABA was eluted with 80% MeCN containing 1% AcOH. MeCN was removed and samples were loaded into 3cc Oasis WAX extraction cartridges (Waters). After washing with water containing 1% AcOH and successively with absolute MeCN, the ABA was eluted using 80% MeCN containing 1% AcOH. For ABA measurements from *Arabidopsis* seedlings, samples (1–20 mg dry weight) were ground with beads in 2-mL tubes and homogenised in 1 mL 80% (v/v) MeCN containing 1% AcOH (v/v) with *d*_6_-ABA. After extracting for 16 h at 4 °C, samples were centrifuged at 4 °C for 10 min at 3,000× *g*, and supernatants were collected. Samples were re-extracted twice with 1 mL 80% MeCN at 4 °C for 30 min, and the supernatants were collected after centrifugation. MeCN was removed with nitrogen gas, and the acidified water extracts were loaded into 1cc Oasis HLB cartridges. After washing with water containing 1% AcOH, plant ABA was eluted with 80% MeCN containing 1% AcOH. MeCN was removed, and samples were loaded into 1cc Bond Elute DEA cartridges (Agilent). After washing with MeOH, the ABA was eluted using MeOH containing 1% AcOH. The fractions containing ABA were dried, dissolved in water containing 1% AcOH, and analysed by LC–MS/MS as described previously^35^. The LC conditions for *Arabidopsis* samples were as follows: solvent A, water containing 0.01% (v/v) acetic acid; solvent B, MeCN containing 0.05% (v/v) acetic acid; and linear gradient, 3 to 40% solvent B over 9 min. Experiments were performed in triplicate, and the results were averaged.

## Additional Information

**How to cite this article**: Takeuchi, J. *et al*. Abscinazole-E3M, a practical inhibitor of abscisic acid 8′-hydroxylase for improving drought tolerance. *Sci. Rep.* **6**, 37060; doi: 10.1038/srep37060 (2016).

**Publisher's note:** Springer Nature remains neutral with regard to jurisdictional claims in published maps and institutional affiliations.

## Supplementary Material

Supplementary Information

## Figures and Tables

**Table 1:** Enzymatic properties of the azole inhibitors of CYP707A.

Compounds	CYP707A^a^		CYP701A^b^
	IC_50_ (nM)	*K*_I_(nM)	IC_50_ (nM)
(+)-Abz-E2B	1,100	—c	1,300
(−)-Abz-E2B	54	38	1,200
(+)-3	1,000	—	1,500
(−)-3	200	—	770
(+)-4	240	—	120
(−)-4	12	—	62
(+)-5	7,700	—	17,000
(−)-5	1,500	—	8,600
(+)-6^d^	1,200	—	1,600
(−)-6	64	35	780

^a^*Arabidopsis* recombinant CYP707A3 expressed in *E. coli*. The inhibition assay for determining the IC_50_ values was performed using 5 μM ABA.

^b^Rice recombinant CYP701A6 expressed in insect cells. The inhibition assay for determining the IC_50_ values was performed using 10 μM *ent*-kaurene.

^c^Not tested.

^d^Compound **6** is also referred to as Abz-E3M.
