# Supplementary material for: Abscinazole-E3M, a practical inhibitor of abscisic acid 8′-hydroxylase for improving drought tolerance
Source: Sci Rep. 2016 Nov 14;6:37060. doi: 10.1038/srep37060 (PMC5107945; doi:10.1038/srep37060)
Supplement: Supplementary Information [file srep37060-s1.pdf]

Supplementary Information

Abscinazole-E3M, a practical inhibitor of abscisic acid 8′-hydroxylase for improving drought tolerance

Jun Takeuchi, Masanori Okamoto, Ryosuke Mega, Yuri Kanno, Mitsunori Seo,
Toshiyuki Ohnishi, Yasushi Todoroki

Supplementary Results

1. Supplementary Figures
2. Supplementary Table
3. Supplementary Note: Chemical synthesis and Characterization
4. Supplementary References

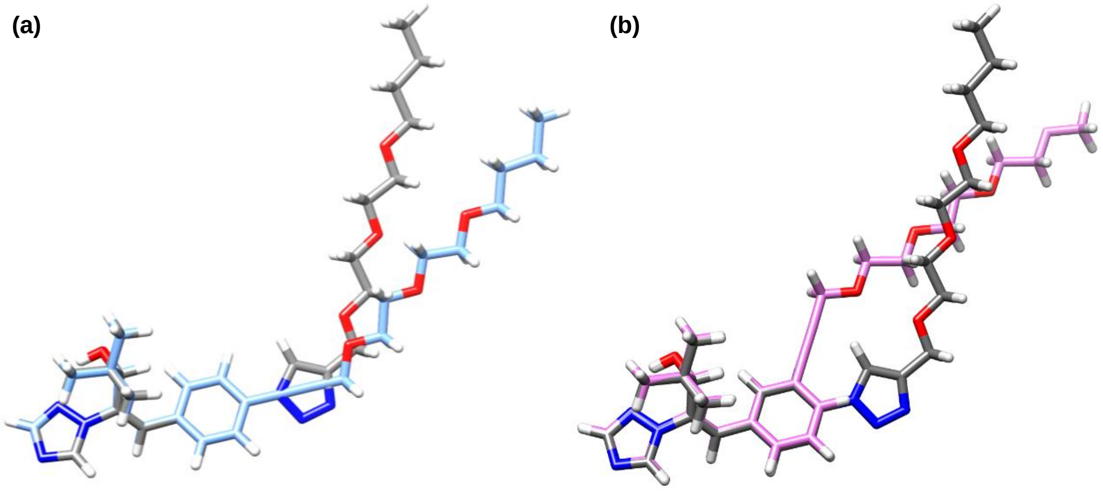

Supplementary Figure 1. The optimized structure of Abz-T compounds. (a) Superposition of compound **3** (cyan) and Abz-E2B (gray). (b) Superposition of compounds **4** (pink) and Abz-E2B (gray). These structures were optimized with B3LYP/6-31G (d).

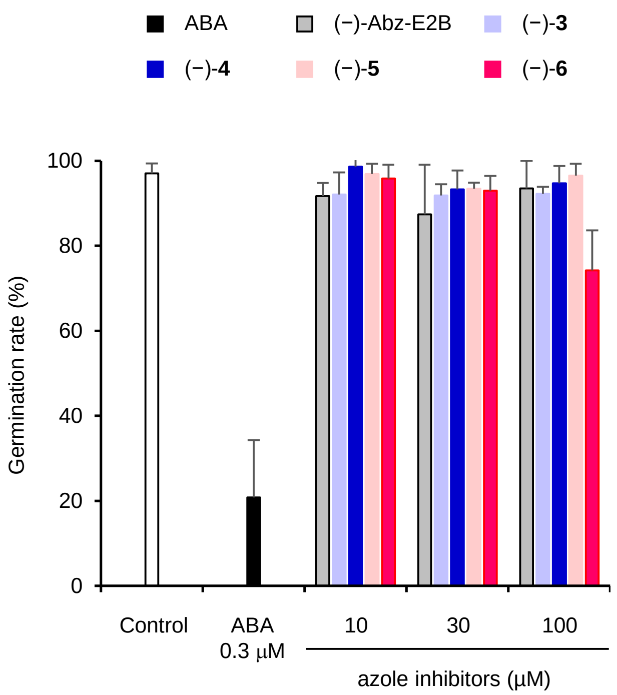

Supplementary Figure 2. Effects of (−)-Abz-T compounds on *Arabidopsis* seed germination. Seed germination rates in the presence of azole inhibitors at 72 h after stratification (n = 3: error bars represent s.d.). Compound **6** is also referred to as Abz-E3M.

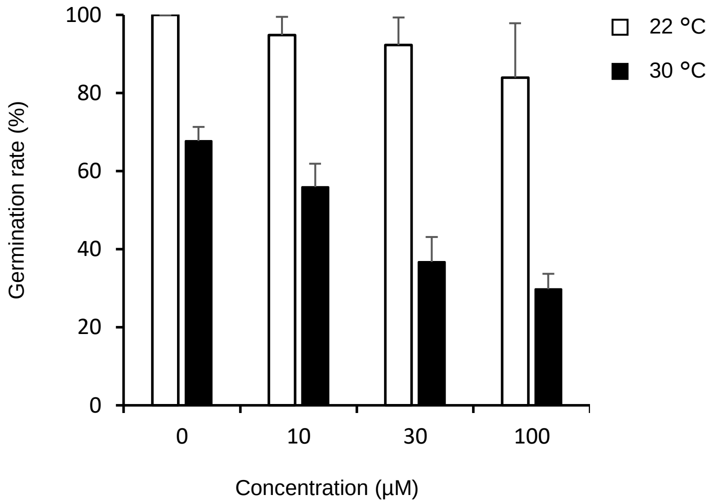

Supplementary Figure 3. Compound 6 enhanced the thermoinduced secondary dormancy in a dose-dependent manner. *Arabidopsis* seeds were incubated with (−)-compound **6** at 22° C or 30° C, and the germination rate was determined at 60 h after stratification (*n* = 3; error bars represent s.d.). Compound **6** is also referred to as Abz-E3M.

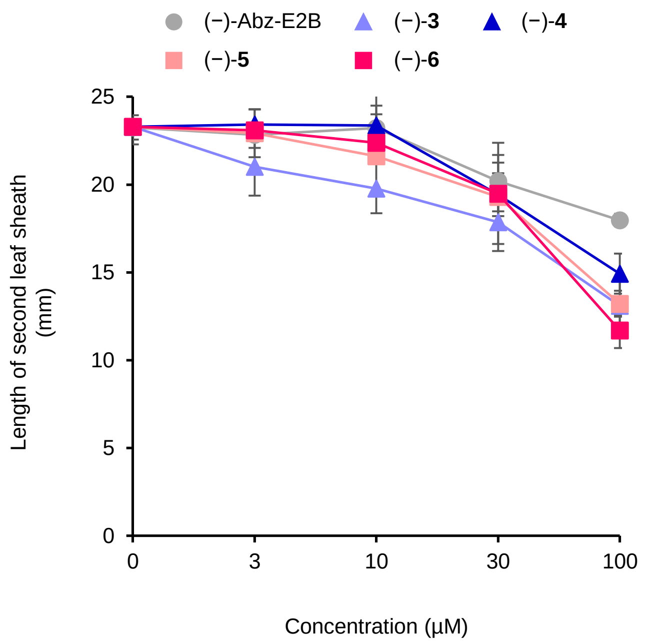

Supplementary Figure 4. Effects of (−)-Abz-T compounds on rice seedling growth. Seedlings grown on test media containing indicated concentration of (−)-Abz-E2B or (−)-Abz-T compounds for 7 d (*n* = 3, error bars represent s.d.). Compound **6** is also referred to as Abz-E3M.

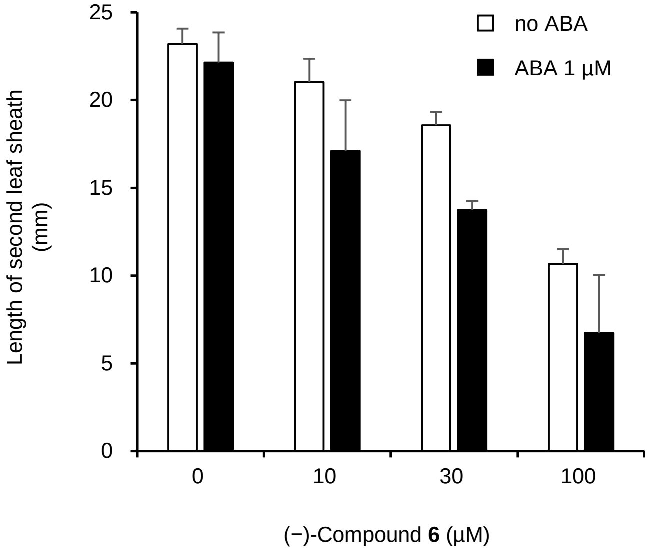

Supplementary Figure 5. (−)-Compound 6 enhanced the effects of ABA on rice seedling growth. Seedlings grown on test media containing indicated concentrations of (−)-compound **6** in the absence or presence of 1 µM ABA. Compound **6** is also referred to as Abz-E3M.

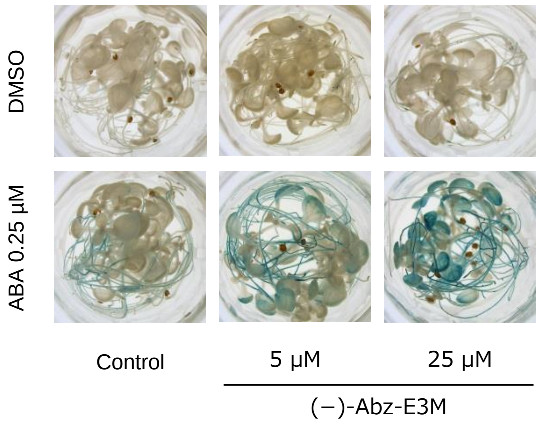

Supplementary Figure 6. (−)-Abz-E3M enhanced the ABA-inducible *MAPKKK18* expression level. Six-day-old seedlings of *MAPKKK18::GUS* reporter-containing transgenic plants were incubated in solutions containing either 5 or 25 μM of (−)-Abz-E3M for 6 h.

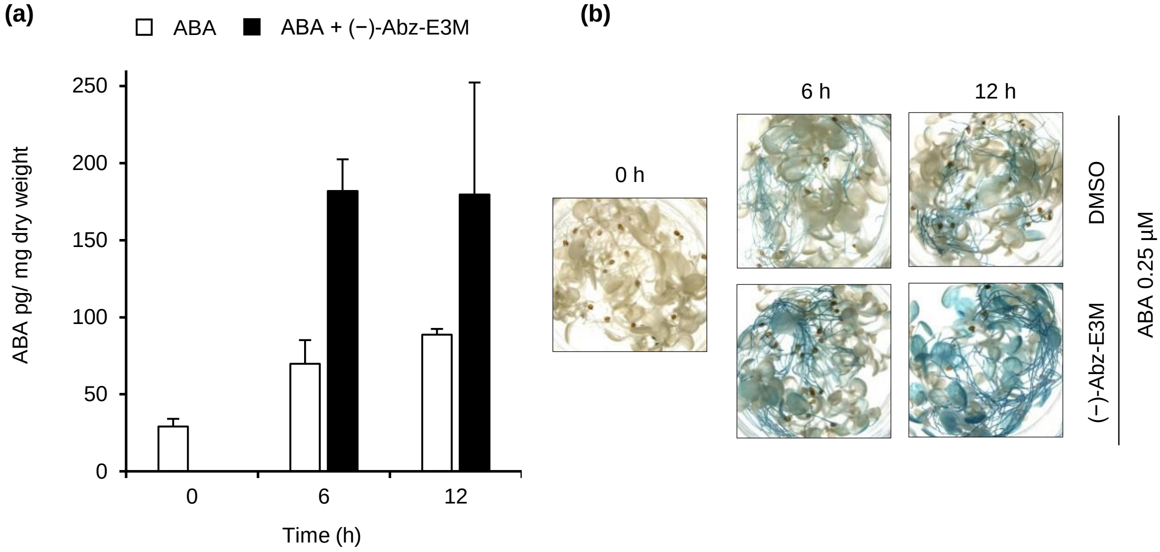

Supplementary Figure 7. Effects of (−)-Abz-E3M on ABA catabolism and the expression level of ABA-responsive gene. (a) Accumulation of ABA after treatment with 0.25 µM ABA in the absence or presence of 25 µ M (−)-Abz-E3M (*n* = 3, error bar represents s.d.). Eleven-day-old seedlings of wild-type (Col accession) were incubated in solutions containing the chemicals for 6 and 12 h. (b) Induction of *MAPKKK18* expression after treatment with 0.25 µM ABA in the absence or presence of 25 µ M (−)-Abz-E3M. Six-day-old seedlings of promoter *MAPKKK18::GUS* reporter transgenic *Arabidopsis* plants were incubated in solutions containing the chemicals for 6 and 12 h.

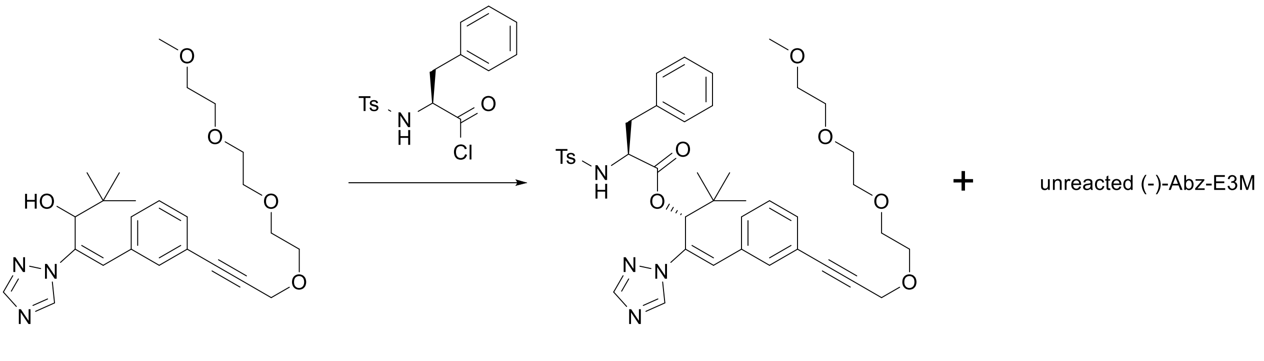

(±)-Abz-E3M

15

Supplementary Figure 8. The facile optical resolution of racemic Abz-E3M. In the reaction, *N*-(*p*-toluenesulfonyl)-L-phenylalanyl chloride preferentially reacted with *R*-(+)-Abz-E3M, and then, we successful isolated unreacted (−)-Abz-E3M in high enantiomeric excess.

Supplementary Table S1: The primer sets for Quantitative RT-PCR (QRT-PCR)

Arabidopssis

	Forward primer	Sequence	Reverse primer	Sequence
Internal control	ACT2F	GAGCAGGAGATGGAAACCTCAAAG	ACT2R	CGATACCTGAGAACATAGTGGTTC
MAPKKK18	MAPKKK18 F	ACGCGCCAGATACTTCTTGGGT	MAPKKK18 R	TCAACCCATTTCGCACACCCGA
RD29A	RD29AF	CAAAGGTGTTTCCTGTCGTGTC	RD29AR	AATCGGTACATCTCTTTTCTCTTCC
RD29B	RD29BF	TATGAATCCTCTGCCGTGAGAGGTG	RD29BR	CAATGGGTTTGGTGTGGTCAGAAGA

Maize

	Forward primer	Sequence	Reverse primer	Sequence
Internal control	ZmACTINF1	GCTACGAGATGCCTGATGGTC	ZmACTINR1	CCCCCACTGAGGACAACG
Zm.12309.1.S1	Zm.12309.1.S1F	ACGCTCAAGGAAGGTTCGTTCCC	Zm.12309.1.S1R	CATCTCCCACTCCGACTTGGCTC
MaizeCOR410	MaizeCOR410F	GCCGATCAGGTGGACGTGAAGGAT	MaizeCOR410R	GCCCTCCTTCTTCTCGCCGACGA
ZmLEA	ZmLEAF	TGCAGCAGGCAGGGGAGAAGG	ZmLEAR	ATGCCGAGCGAGTTCATCATCG

Supplementary Note: Chemical Synthesis and Characterization

General procedures

(+)-ABA was a gift from Toray Industries Inc., Tokyo, Japan. ^{1}H NMR spectra were recorded with tetramethylsilane as the internal standard using JEOLJNM-EX270 (270 MHz) and JNM-LA500 (500 MHz) NMR spectrometers (JEOL Ltd., Tokyo, Japan). ^{13}C NMR and 2D-correlation NMR experiments were recorded using a JNM-LA500 (500 MHz) NMR spectrometer (JEOL Ltd.). All peak assignments refer to the numbering in structure **UNI** (Fig. 1). High resolution mass spectra were obtained with a JEOL JMS-T100LC AccuTOF mass spectrometer (ESI-TOF, positive mode; JEOL Ltd.). Optical rotations were recorded with a Jasco DIP-1000 digital polarimeter. Column chromatography was performed using silica gel (Wakosil C-200, Wako Pure Chemical Industries, Ltd., Osaka, Japan).

1-(4-iodophenyl)-4,4-dimethyl-2-(1H-1,2,4-triazol-1-yl)pent-1-en-3-one (8)

To a stirred solution of **7**[1] (105 mg, 0.63 mmol) in Ac_2O (1 mL) was added K_2CO_3 (87 mg, 0.63 mmol) and 4-iodobenzaldehyde (146 mg, 0.63 mmol) at room temperature. The mixture was stirred for 5 h at 100 °C. After quenching with water (6 mL), it was extracted with EtOAc (16 mL × 3). The organic layer was washed with brine, dried over Na_2SO_4, and concentrated *in vacuo*. The residual oil was purified by silica gel chromatography with 20% EtOAc in hexane to obtain a mixture of **8*Z*** and **8*E*** (102 mg, 43%) as a white solid.

8*Z*: ^{1}H NMR (270 MHz, CD_3OD, TMS): δ 0.99 (9H, s, *t*-butyl), 7.13 (1H, m, H-2'' or 6''), 7.16 (1H, m, H-2'' or 6''), 7.24 (1H, br s, H-1), 7.73 (1H, m, H-3'' or 5''), 7.77 (1H, m, H-3'' or 5''), 8.12 (1H, s, H-3'), 8.92 (1H, s, H-5'); HRMS (ESI-TOF, positive mode): calcd for $C_{15}H_{16}IN_3NaO$ $[M+Na]^+$ 404.0235, found 404.0230.

8*E*: ^{1}H NMR (270 MHz, CD_3OD, TMS): δ 1.26 (9H, s, *t*-butyl), 6.62 (1H, m, H-2'' or 6''), 6.66 (1H, m, H-2'' or 6''), 7.64 (1H, m, H-3'' or 5''), 7.68 (1H, m, H-3'' or 5''), 7.73 (1H, br s, H-1), 8.23 (1H, s, H-3'), 8.45 (1H, s, H-5'); HRMS (ESI-TOF, positive mode): calcd for C15H16IN3NaO [M+Na]+ 404.0235, found 404.0229.

(*E*)-1-(4-iodophenyl)-4,4-dimethyl-2-(1*H*-1,2,4-triazol-1-yl)pent-1-en-3-ol (10)

The solution of a mixture of **8*E*** and **8*Z*** (84 mg, 0.21 mmol) in MeOH was irradiated with UV light (365 nm, UVP B-100A) for 1.5 h at 0 °C. Evaporation of solvent *in vacuo* yielded the crude product **8*E***, as a white solid (80 mg). To a solution of **8*E*** (80 mg, 0.21 mmol) in MeOH (2 mL) was added $NaBH_4$ (22 mg, 0.58 mmol) at 0 °C. The mixture was stirred for a further 20 min at 0 °C and then the ice bath was removed. The reaction mixture was stirred at room temperature for 3 h. After quenching with water (3 mL), it was extracted with EtOAc (18 mL × 3). The organic layer was

washed with brine, dried over Na_2SO_4, and concentrated *in vacuo*. The residual oil was purified by silica gel chromatography with 30% EtOAc in hexane to obtain **10** (61 mg, 73%) as a white solid. ^{1}H NMR (270 MHz, CD_3OD, TMS): δ 0.56 (9H, s, *t*-butyl), 4.54 (1H, s, H-3), 6.95 (1H, s, H-1), 7.09 (1H, m, H-2" or 6"), 7.12 (1H, m, H-2" or 6"), 7.68 (1H, m, H-3" or 5"), 7.70 (1H, m, H-3" or 5"), 7.99 (1H, s, H-3'), 8.82 (1H, s, H-5'); HRMS (ESI-TOF, positive mode): calcd for $C_{15}H_{19}IN_3O$ $[M+Na]^+$ 384.0573, found 384.0571.

(*E*)-2-(2-((3-(4-(3-hydroxy-4,4-dimethyl-2-(1*H*-1,2,4-triazol-1-yl)pent-1-en-1-yl)phenyl)prop-2-yn-1-yl)oxy)ethoxy)ethyl 4-methylbenzenesulfonate (13)

To a stirred solution of **10** (31 mg, 82 μmol) in THF (0.5 mL) was successively added trimethylamine (22 μL, 160 μmol), CuI (2.4 mg, 13 μmol) and *trans*-dichlorobis(triphenylphosphine)Pd(II) 1.6 mg (2.3 μmol). After being stirred for 30 min at room temperature, a solution of **12**[2] (38 mg, 130 μmol) in THF (0.2 mL) was added to the stirred mixture. The reaction mixture was stirred for 90 min at room temperature, and then it was filtered through silica gel (EtOAc). The filtrate was successively washed with water and brine, dried over Na_2SO_4, and concentrated *in vacuo*. The residual oil was purified by silica gel chromatography with 30% EtOAc in hexane to obtain **13** (68 mg, 84%) as a yellow oil. ^{1}H NMR (270 MHz, $CDCl_3$, TMS): δ 0.66 (9H, s, *t*-butyl), 2.44 (3H, s, $-OSO_2Ph\mathit{CH_3}$), 3.64–3.74 (6H, m, $-O(\mathit{CH_2})_2O-$ and $-O\mathit{CH_2}CH_2OSO_2Ph$), 4.19 (2H, t, *J*=4.6 Hz, $-OCH_2\mathit{CH_2}OSO_2Ph$), 4.41 (2H, s, $-C{\equiv}C\mathit{CH_2}-$), 4.58 (1H, br s, H-3), 6.93 (1H, s, H-1), 7.34 (4H, m, H-2", 6" and $-OSO_2\mathit{Ph}CH_3$), 7.49 (2H, m, H-3" and 5"), 7.81 (2H, m, $-OSO_2\mathit{Ph}CH_3$), 8.06 (1H, br s, H-3'), 8.49 (1H, br s, H-5'); HRMS (ESI-TOF, positive mode): calcd for $C_{29}H_{35}N_3NaO_6S$ $[M+Na]^+$ 576.2144, found 576.2142.

(*E*)-1-(4-(3-(2-(2-butoxyethoxy)ethoxy)prop-1-yn-1-yl)phenyl)-4,4-dimethyl-2-(1*H*-1,2,4-triazol-1-yl)pent-1-en-3-ol (3)

To a stirred solution of 1-butanol (0.5 mL) was added NaH (60% in oil, 22 mg, 0.54 mmol) at room temperature under an atmosphere of Ar. After being stirred for 5 min, a solution of **13** (22 mg, 39 μmol) in 1-butanol (0.3 mL) was added to the stirred mixture. The reaction mixture was stirred for 11 h at room temperature. After quenching with sat. NH_4Cl (3 mL), it was diluted with water (5 mL) and extracted with EtOAc (8 mL × 3). The organic layer was washed with brine, dried over Na_2SO_4, and concentrated *in vacuo*. The residual oil was purified by silica gel chromatography with 30% EtOAc in hexane to obtain **3** (14.5 mg, 82%) as a pale yellow oil (six steps, 15% overall yield). ^{1}H NMR (500 MHz, $CDCl_3$, TMS): δ 0.22 (9H, s, *t*-butyl), 0.48 (3H, t, *J*=7.6 Hz, H-11'"), 0.90-0.98 (2H, m, H-10'"), 1.08-1.14 (2H, m, H-9'"), 3.04 (2H, t, *J*=6.7 Hz, H-8'"), 3.14-3.16 (2H, m, H-7'"), 3.20-3.22 (2H, m, H-6'"), 3.25-3.26 (2H, m, H-5'"), 3.31-3.33 (2H, m, H-4'"), 4.00 (2H, s, H-3'"), 4.24 (1H, s, H-3), 6.66 (1H, s, H-1), 6.97-6.98 (2H, m, H-2" and 6"), 7.05-7.06 (2H, m, H-3" and 5"),

7.65 (1H, s, H-3'), 8.48 (1H, s, H-5'); ^{13}C NMR (125 MHz, $CDCl_3$): δ 14.2 (C-11'''), 20.3 (C-10'''), 26.6 (*t*-butyl), 32.9 (C-9'''), 37.0 (C-4), 59.7 (C-3'''), 70.3 (C-4'''), 71.2 (C-7'''), 71.5 (C-5''' or 6'''), 71.6 (C-5''' or 6'''), 72.1 (C-8'''), 75.4 (C-3), 86.6 (C-1'''), 87.2 (C-2'''), 123.7 (C-4''), 130.1 (C-2'' and 6''), 130.2 (C-1), 132.9 (C-3'' and 5''), 136.2 (C-1''), 139.7 (C-2), 146.0 (C-5'), 151.5 (C-3'); UV λ_{max} (MeOH) nm (ε): 271 (24000); HRMS (ESI-TOF, positive mode): calcd for $C_{26}H_{37}N_3NaO_4$ $[M+Na]^+$ 478.2682, found 478.2676.

A Chiralcel OD HPLC column (250 × 10.0 mm i.d., Daicel; solvent, 8% 2-propanal in hexane; flow rate, 4.5 ml/min; detection, 254 nm) was injected with (±)-**3**. The material at t_R 23.4 and 32.8 min were collected to give (−)-**3** (11.8 mg) and the (+)-enantiomer (11.2 mg) with an optical purity of 99.9% and 99.9%, respectively. (−)-**3**: $[\alpha]_D^{27}$ −36 (MeOH; *c* 0.75). (+)-**3**: $[\alpha]_D^{28}$ +32 (MeOH; *c* 0.78).

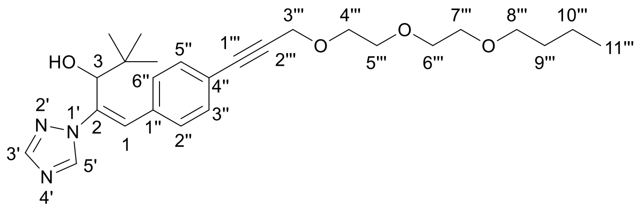

(*E*)-1-(4-(2,5,8,11-tetraoxatetradec-13-yn-14-yl)phenyl)-4,4-dimethyl-2-(1*H*-1,2,4-triazol-1-yl)pent-1-en-3-ol (5)

To a stirred solution of 2-methoxyehanol (2 mL) was added NaH (60% in oil, 76 mg, 1.9 mmol) at room temperature under an atmosphere of Ar. After being stirred for 20 min, a solution of **13** (87 mg, 160 μmol) in 2-methoxyehanol (1.0 mL) was added to the stirred mixture. The reaction mixture was stirred for 7.5 h at 60 °C. After quenching with sat. NH_4Cl (10 mL), it was diluted with water (10 mL) and extracted with EtOAc (30 mL × 3). The organic layer was washed with brine, dried over Na_2SO_4, and concentrated *in vacuo*. The residual oil was purified by silica gel chromatography with 5% MeOH in CH_2Cl_2 to obtain **5** (61 mg, 85%) as a pale yellow oil (six steps, 16% overall yield), which was further purified for bioassays by HPLC (YMC Hydrosphere C18, 150 × 20.0 mm i.d.; solvent, 65% MeOH; flow rate, 8 ml/min; detection, 254 nm) to obtain a colorless oil. ^{1}H NMR (500 MHz, $CDCl_3$, TMS): δ 0.66 (9H, s, *t*-butyl), 3.38 (3H, s, H-10'''), 3.54-3.56 (2H, m, H-9'''), 3.65-3.66 (2H, m, H-8'''), 3.67-3.71 (4H, m, H-6''' and 7'''), 3.71-3.74 (2H, m, H-5'''), 3.77-3.79 (2H, m, H-4'''), 4.28 (1H, br d, *J*=8.5 Hz, O*H*), 4.44 (2H, s, H-3'''), 4.58 (1H, d, *J*=8.5 Hz, H-3), 6.93 (1H, s, H-1), 7.33 (2H, d, *J*=8.3 Hz, H-2'' and 6''), 7.48 (2H, d, *J*=8.3 Hz, H-3'' and 5''), 8.04 (1H, s, H-3'), 8.49 (1H, s, H-5'); ^{13}C NMR (125 MHz, $CDCl_3$): δ 26.0 (*t*-butyl), 36.1 (C-4), 59.0 (C-10'''), 59.1 (C-3'''), 69.2 (C-4'''), 70.45 (C-5''', 6''', 7''' or 8'''), 70.51 (C-5''', 6''', 7''' or 8'''), 70.59 (C-5''', 6''', 7''' or 8'''), 70.63 (C-5''', 6''', 7''' or 8'''), 71.9 (C-9'''), 75.7 (C-3), 85.6 (C-1'''), 86.5 (C-2'''), 122.7 (C-4''), 128.4 (C-1), 128.7 (C-2'' and 6''), 132.0 (C-3'' and 5''), 133.8 (C-1''), 137.1 (C-2), 143.1 (C-5'), 151.5 (C-3'); UV λ_{max} (MeOH) nm (ε): 270 (24000); HRMS (ESI-TOF, positive mode): calcd for $C_{25}H_{35}N_3NaO_5$

$[M+Na]^+$ 480.2474, found 480.2473.

A Chiralcel OD HPLC column (250 × 10.0 mm i.d., Daicel; solvent, 15% 2-propanal in hexane; flow rate, 4.5 ml/min; detection, 254 nm) was injected with (±)-**5**. The material at t_R 19.2 and 25.6 min were collected to give (−)-**5** (8.1 mg) and the (+)-enantiomer (8.2 mg) with an optical purity of 99.8% and 99.8%, respectively. (−)-**5**: $[\alpha]_D^{32}$ −35 (MeOH; *c* 0.81). (+)-**5**: $[\alpha]_D^{32}$ +36 (MeOH; *c* 0.82).

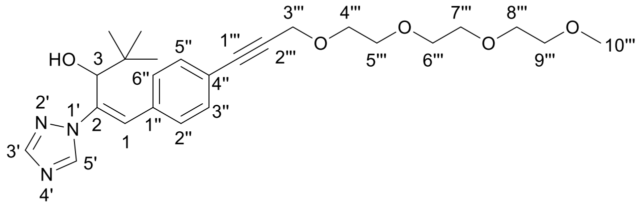

1-(3-iodophenyl)-4,4-dimethyl-2-(1H-1,2,4-triazol-1-yl)pent-1-en-3-one (9)

To a stirred solution of 3-iodobenzaldehyde (9.04 g, 39.0 mmol) in Ac_2O (16 mL) was added K_2CO_3 (5.39 g, 0.39 mmol). A solution of **7** (6.51 g, 38.9 mmol) in Ac_2O (21 mL) was then added dropwise to the stirred mixture. The mixture was stirred for 7 h at 100 °C and then was cooled to room temperature. After quenching with water (150 mL), it was extracted with EtOAc (150 mL × 3). The organic layer was washed with brine, dried over Na_2SO_4, and concentrated *in vacuo*. The residual oil was purified by silica gel chromatography with hexane-EtOAc stepwise to obtain a mixture (1:1, based on TLC) of **9*Z*** and **9*E*** (8.88g, 60%) as a yellow oil.

(*E*)-1-(3-iodophenyl)-4,4-dimethyl-2-(1H-1,2,4-triazol-1-yl)pent-1-en-3-one (9*E*)

The solution of a mixture of **9*Z*** and **9*E*** (8.88 g, 23.3 mmol) in MeOH (100 mL) was irradiate with UV light (365 nm, UVP B-100A) for 2 h at 0 °C. After concentration *in vacuo*, the residual oil was purified by silica gel chromatography with hexane-EtOAc stepwise to obtain **9*E*** (7.26 g, 83%) as a yellow oil. ^1H NMR (500 MHz, $CDCl_3$, TMS): δ 1.03 (9H, s, *t*-butyl), 7.08 (1H, s, H-1), 7.10 (1H, br t, *J*=7.9 Hz, H-5"), 7.31 (1H, m, H-6"), 7.67-7.69 (2H, m, H-2" and 4"), 8.06 (1H, s, H-3'), 8.26 (1H, s, H-5'); ^{13}C NMR (125 MHz, $CDCl_3$): δ 27.1, 45.5, 94.5, 120.4, 128.1, 130.4, 134.5, 134.8, 137.6, 138.0, 141.8, 152.7, 208.0; HRMS (ESI-TOF, positive mode): calcd for $C_{15}H_{16}IN_3NaO$ $[M+Na]^+$ 404.0236, found 404.0240.

(*E*)-1-(3-iodophenyl)-4,4-dimethyl-2-(1H-1,2,4-triazol-1-yl)pent-1-en-3-ol (11)

To a stirred solution of **9*E*** (6.54 g, 17.2 mmol) in MeOH (180 mL) was added $NaBH_4$ (0.91 g, 24.1 mmol) at 0 °C. The mixture was stirred for 3 h at room temperature. After quenching with water (300 mL), it was diluted with water (500 mL) and extracted with EtOAc (600 mL × 3). The organic layer was washed with brine, dried over Na_2SO_4, and concentrated *in vacuo*. The residual oil was purified by silica gel chromatography with hexane-EtOAc stepwise to obtain **11** (6.15g, 94%) as a

white solid. ^{1}H-NMR (500 MHz, $CDCl_3$, TMS): δ 0.68 (9H, s, *t*-butyl), 4.30 (1H, d, *J*=8.5 Hz, O*H*), 4.54 (1H, d, *J*=8.5 Hz, H-3), 6.88 (1H, s, H-1), 7.15 (1H, t, *J*=7.6 Hz, H-5''), 7.35 (1H, d, *J*=7.6 Hz, H-6''), 7.69 (1H, d, *J*=7.6 Hz, H-4''), 7.72 (1H, br s, H-2''), 8.04 (1H, s, H-3'), 8.50 (1H, s, H-5'); ^{13}C NMR (125 MHz, $CDCl_3$): δ 26.1, 36.2, 75.6, 94.4, 127.4, 128.0, 130.3, 135.9, 137.2, 137.4, 137.6, 143.2, 151.6; HRMS (ESI-TOF, positive mode): calcd for $C_{15}H_{18}IN_3NaO$ $[M+Na]^+$ 406.0392, found 406.0391.

(*E*)-2-(2-((3-(3-(3-hydroxy-4,4-dimethyl-2-(1H-1,2,4-triazol-1-yl)pent-1-en-1-yl)phenyl)prop-2-yn-1-yl)oxy)ethoxy)ethyl 4-methylbenzenesulfonate (14)

To a stirred solution of **11** (6.15 g, 16.0 mmol) in THF (100 mL) was successively added trimethylamine (11.2 mL, 80.8 mmol), CuI (280 mg, 1.47 mmol) and *trans*-dichlorobis(triphenylphosphine)Pd(II) (29.0 mg (0.413 mmol) under an atmosphere of Ar. After being stirred for 30 min at room temperature, a solution of **12** (5.31 g, 17.8 mmol) in THF (70 mL) was added to the stirred mixture. The reaction mixture was stirred for 2 h at room temperature, and then it was filtered through silica gel (EtOAc). The filtrate was successively washed with water and brine, dried over Na_2SO_4, and concentrated *in vacuo*. The residual oil was purified by silica gel chromatography with hexane-EtOAc stepwise to obtain **14** (6.05 g, 69%) as a pale yellow oil. ^{1}H NMR (500 MHz, $CDCl_3$, TMS): δ 0.66 (9H, s, *t*-butyl), 2.43 (3H, s, -OSO_2PhCH_3), 3.65-3.73 (6H, m, -O(CH_2)$_2$O- and -O$CH_2CH_2OSO_2$Ph), 4.18-4.20 (2H, m, -$OCH_2CH_2OSO_2$Ph), 4.25 (1H, d, *J*=8.5 Hz, OH), 4.40 (2H, s, -C≡CCH_2-), 4.54 (1H, d, *J*=8.5 Hz, H-3), 6.92 (1H, s, H-1), 7.33-7.34 (3H, m, H-6'' and -OSO_2*Ph*CH_3), 7.37 (1H, t, *J*=7.6 Hz, H-5''), 7.43 (1H, d, *J*=7.6 Hz, H-4''), 7.46 (1H, br s, H-2''), 7.80 (2H, m, -OSO_2*Ph*CH_3), 8.05 (1H, s, H-3'), 8.51 (1H, s, H-5'); ^{13}C NMR (125 MHz, $CDCl_3$): δ 21.6, 26.1, 36.2, 59.2, 68.7, 69.2, 69.2, 70.6, 75.7, 85.7, 85.9, 123.3, 128.0, 128.0, 128.1, 128.8, 128.8, 129.8 129.8, 131.5, 131.8, 133.0, 134.1, 137.3, 143.2, 144.8, 151.6; HRMS (ESI-TOF, positive mode): calcd for $C_{29}H_{35}N_3NaO_6S$ $[M+Na]^+$ 576.2144, found 576.2148.

(*E*)-1-(3-(3-(2-(2-butoxyethoxy)ethoxy)prop-1-yn-1-yl)phenyl)-4,4-dimethyl-2-(1*H*-1,2,4-triazol-1-yl)pent-1-en-3-ol (4)

To a stirred solution of 1-butanol (0.5 mL) was added NaH (60% in oil, 13 mg, 0.32 mmol) at room temperature under an atmosphere of Ar. After being stirred for 15 min, a solution of **14** (15 mg, 27 μmol) in 1-butanol (1.5 mL) was added to the stirred mixture. The reaction mixture was stirred for 100 min at 60 °C. After quenching with sat. NH_4Cl (3 mL), it was diluted with water (3 mL) and extracted with EtOAc (10 mL × 3). The organic layer was washed with brine, dried over Na_2SO_4, and concentrated *in vacuo*. The residual oil was purified by silica gel chromatography with 30% EtOAc in hexane to obtain **4** (10.8 mg, 88%) as a colorless oil (six steps, 28% overall yield), which was further purified for bioassays by HPLC (YMC Hydrosphere C18, 150 × 20.0 mm i.d.; solvent,

75% MeOH; flow rate, 9 ml/min; detection, 254 nm) to obtain a colorless oil. ^{1}H NMR (500 MHz, $CDCl_3$, TMS): δ 0.66 (9H, s, *t*-butyl), 0.91 (3H, t, *J*=7.3 Hz, H-11'''), 1.34-1.38 (2H, m, H-10'''), 1.54-1.60 (2H, m, H-9'''), 3.47 (2H, t, *J*=7.0 Hz, H-8'''), 3.60-3.62 (2H, m, H-7'''), 3.67-3.69 (2H, m, H-6'''), 3.72-3.74 (2H, m, H-5'''), 3.77-3.79 (2H, m, H-4'''), 4.24 (1H, d, *J*=8.5 Hz, O*H*), 4.44 (2H, s, H-3'''), 4.55 (1H, d, *J*=8.5 Hz, H-3), 6.92 (1H, s, H-1), 7.34-7.38 (2H, m, H-5'' and 6''), 7.42-7.45 (2H, m, H-2'' and 4''), 8.05 (1H, s, H-3'), 8.49 (1H, s, H-5'); ^{13}C NMR (125 MHz, $CDCl_3$): δ 13.9 (C-11'''), 19.3 (C-10'''), 26.1 (*t*-Bu), 31.7 (C-9'''), 36.2 (C-4), 59.2 (C-3'''), 69.3 (C-4'''), 70.1 (C-5''', 6''' or 7'''), 70.5 (C-5''', 6''' or 7'''), 70.7 (C-5''', 6''' or 7'''), 71.2 (C-8'''), 75.7 (C-3), 85.5 (C-1'''), 86.1 (C-2'''), 123.4 (C-3''), 128.1 (C-1), 128.7 (C-5'' or 6''), 128.8 (C-5'' or 6''), 131.5 (C-4''), 134.0 (C-1''), 137.3 (C-2), 143.1 (C-5'), 151.6 (C-3'); UV λ_{max} (MeOH) nm (ε): 241 (35000); HRMS (ESI-TOF, positive mode): calcd for $C_{26}H_{37}N_3NaO_4$ $[M+Na]^+$ 478.2682, found 478.2675.

A Chiralcel OD HPLC column (250 × 10.0 mm i.d., Daicel; solvent, 10% 2-propanal in hexane; flow rate, 4.5 ml/min; detection, 254 nm) was injected with (±)-**4**. The material at t_R 16.4 and 24.5 min were collected to give (−)-**4** (7.0 mg) and the (+)-enantiomer (7.0 mg) with an optical purity of 99.9% and 99.8%, respectively. (−)-**4**: $[\alpha]_D^{32}$ −4.4 (MeOH; *c* 0.5). (+)-**4**: $[\alpha]_D^{32}$ +3.8 (MeOH; *c* 0.5).

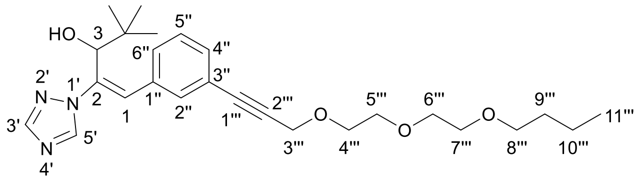

(*E*)-1-(3-(2,5,8,11-tetraoxatetradec-13-yn-14-yl)phenyl)-4,4-dimethyl-2-(1*H*-1,2,4-triazol-1-yl)pent-1-en-3-ol, Abz-E3M (6)

To a stirred solution of 2-methoxyehanol (45 mL, 2.3 mmol) was added NaH (60% in oil, 4.86 g, 122 mmol) at 0 °C under an atmosphere of Ar. After being stirred for 15 min, a solution of **14** (6.08 g, 11.0 mmol) in 2-methoxyethanol (135 mL) was added to the stirred mixture at the same temperature. The reaction mixture was stirred for 30 min at room temperature and then this reaction mixture was stirred for 1 h at 80 °C. After quenching with sat. NH_4Cl (300 mL) at 0 °C, it was diluted with water (400 mL) and extracted with EtOAc (600 mL × 3). The organic layer was washed with brine, dried over Na_2SO_4, and concentrated *in vacuo*. The residual oil was purified by silica gel chromatography with 5% MeOH in EtOAc to obtain **6** (4.28 g, 85%) as a pale yellow oil (six steps, 27% overall yield), which was further purified for bioassays by HPLC (YMC Hydrosphere C18, 150 × 20.0 mm i.d.; solvent, 70% MeOH; flow rate, 8 ml/min; detection, 254 nm) to obtain a colorless oil. ^{1}H NMR (500 MHz, $CDCl_3$, TMS): δ 0.66 (9H, s, *t*-butyl), 3.38 (3H, s, H-10'''), 3.55-3.56 (2H, m, H-9'''), 3.65-3.73 (8H, m, H-5''', 6''', 7''' and 8'''), 3.77-3.39 (2H, m, H-4'''), 4.34 (1H, d, *J*=8.5 Hz, O*H*), 4.44 (2H, s, H-3'''), 4.56 (1H, d, *J*=8.5 Hz, H-3), 6.93 (1H, s, H-1), 7.33 (1H, d, *J*=7.6 Hz, H-6''), 7.36 (1H, t, *J*=7.6 Hz, H-5''), 7.42 (1H, d, *J*=7.6 Hz, H-4''), 7.46 (1H, br s, H-2''), 8.04 (1H, s, H-3'), 8.54 (1H, s,

H-5'); ^{13}C NMR (125 MHz, $CDCl_3$): δ 26.1 (*t*-butyl), 36.1 (C-4), 59.0 (C-10'''), 59.1 (C-3'''), 69.2 (C-4'''), 70.44 (C-5''', 6''', 7''' or 8'''), 70.49 (C-5''', 6''', 7''' or 8'''), 70.56 (C-5''', 6''', 7''' or 8'''), 70.61 (C-5''', 6''', 7''' or 8'''), 71.9 (C-9'''), 75.6 (C-3), 85.6 (C-1''' or 2'''), 86.0 (C-1''' or 2'''), 123.3 (C-3''), 128.0 (C-1), 128.74 (C-5'' or 6''), 128.76 (C-5'' or 6''), 131.4 (C-4''), 131.8 (C-2''), 134.0 (C-1''), 137.4 (C-2), 143.2 (C-5'), 151.5 (C-3'); UV λ_{max} (MeOH) nm (ε): 241 (33000); HRMS (ESI-TOF, positive mode): calcd for $C_{25}H_{35}N_3NaO_5$ $[M+Na]^+$ 480.2474, found 480.2470.

A Chiralcel OD HPLC column (250 × 10.0 mm i.d., Daicel; solvent, 18% 2-propanal in hexane; flow rate, 4.5 ml/min; detection, 254 nm) was injected with (±)-**6**. The material at t_R 14.9 and 23.5min were collected to give (−)-**6** (5.2 mg) and the (+)-enantiomer (5.4 mg) with an optical purity of 99.9% and 99.9%, respectively. (−)-**6**: $[\alpha]_D^{29}$ −2.8 (MeOH; *c* 0.52). (+)-**6**: $[\alpha]_D^{30}$ +3.6 (MeOH; *c* 0.54).

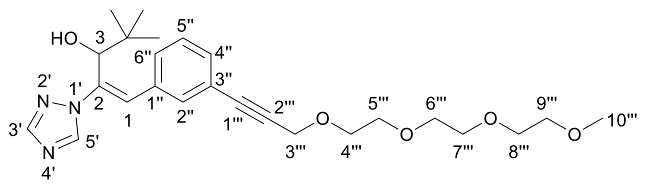

R-MTPA-esters of (+)-Abz-E3M

To a stirred solution of **(+)-Abz-E3M** (19.8 mg, 44 µmol) in dry pyridine-CH_2Cl_2 (1:1, 400 µL) was added 4-dimethylaminopyridene (23.6 mg, 210 µmol) and *S*-α-methoxy-α-(trifluoromethyl)phenylacetyl chloride (MTPA-Cl) (32 µL, 172 µmol) under an atmosphere of Ar. After stirred for 21 h at room temperature, sat. NH_4Cl (2 mL) was added to quench the reaction. The resulting mixture was extracted with EtOAc (10 mL × 3). The organic layer was washed with brine, dried over Na_2SO_4, and concentrated *in vacuo*. The residual oil was purified by silica gel chromatography with 25% EtOAc in hexane to obtain ***R*-MTPA-(+)-Abz-E3M** (26.6 mg, 91%) as a colorless oil, which was further purified by silicagel HPLC (YMC-Pack SIL-06, 150 × 20.0 mm i.d.; solvent, 90% EtOAc in hexane; flow rate, 7 ml/min; detection, 254 nm) to obtain a colorless oil. ^1H NMR (270 MHz, $CDCl_3$, TMS): δ 0.65 (9H, s, *t*-butyl), 3.38 (3H, s, H-10'''), 3.50-3.80 (15H, m, H-4''', 5''', 6''', 7''',8''', 9''' and O*Me*), 4.45 (2H, s, H-3'''), 6.19 (1H, s, H-3), 7.24 (1H, s, H-1), 7.41-7.60 (9H, m, H-2'', 4'', 5'', 6'' and COC*Ph*), 7.97 (1H, s, H-3'), 8.05 (1H, s, H-5'); HRMS (ESI-TOF, positive mode): calcd for $C_{35}H_{42}F_3N_3NaO_7$ $[M+Na]^+$ 696.2873, found 696.2874.

***R*-MTPA-esters of (−)-Abz-E3M**

To a stirred solution of **(−)-Abz-E3M** (4.5 mg, 9.8 µmol) in dry pyridine-CH_2Cl_2 (1:1, 400 µL) was added 4-dimethylaminopyridene (6.1 mg, 50 µmol) and *S*-MTPA-Cl (16 µL, 86 µmol) under an atmosphere of Ar. After stirred for 150 h at 75 °C, sat. NH_4Cl (2 mL) was added to quench the reaction. The resulting mixture was extracted with EtOAc (8 mL × 3). The organic layer was washed with brine, dried over Na_2SO_4, and concentrated *in vacuo*. The residual oil was purified by silica gel

chromatography with 25% EtOAc in hexane, and then it was further purified by silicagel HPLC (YMC-Pack SIL-06, 150 × 20.0 mm i.d.; solvent, 80% EtOAc in hexane; flow rate, 10 ml/min; detection, 254 nm) to obtain ***R*-MTPA-(−)-Abz-E3M** (1.2 mg, 18%) as a colorless oil. ^{1}H NMR (270 MHz, $CDCl_3$, TMS): δ 0.72 (9H, s, *t*-butyl), 3.38 (3H, s, H-10'''), 3.49-3.81 (15H, m, H-4''', 5''', 6''', 7''',8''', 9''' and O*Me*), 4.45 (2H, s, H-3'''), 6.13 (1H, s, H-3), 7.14 (1H, s, H-1), 7.39-7.65 (9H, m, H-2'', 4'', 5'', 6'' and COC*Ph*), 7.77 (1H, s, H-3'), 7.92 (1H, s, H-5'); HRMS (ESI-TOF, positive mode): calcd for $C_{35}H_{42}F_3N_3NaO_7$ $[M+Na]^+$ 696.2873, found 696.2868.

Optical resolution of Abz-E3M using a chiral derivatizing agent

To a stirred solution of **(±)-Abz-E3M** (52.7 mg, 0.115 mmol) in toluene (5 mL) was added *N*-(*p*-toluenesulfonyl)-L-phenylalanyl chloride (195 mg, 0.577 mmol) under an atmosphere of Ar. The mixture was stirred for 4 h at 140 °C. After cooling to cooling down to room temperature, it was quenched with sat. $NaHCO_3$ (12 mL) and extracted with EtOAc (15 mL × 3). The organic layer washed with brine, dried over dried over Na_2SO_4, and concentrated *in vacuo*. The residual oil was purified by silica gel chromatography with hexane-EtOAc stepwise to obtain unreacted ***S*-(−)-Abz-E3M** (18.1 mg, 34%) with an optical purity of 93.9% and, **(*E*)-1-(3-(2,5,8,11-tetraoxatetradec-13-yn-14-yl)phenyl)-4,4-dimethyl-2-(1H-1,2,4-triazol-1-yl)pent-1-en-3-yl tosyl-L-phenylalaninate (15)** as a yellow oil (53.9 mg, 59%).

15: ^{1}H-NMR (270 MHz, CD_3OD, TMS): δ 0.52 (9H, s, H_3-5, 6 and 7), 2.23 (3H, s, $NHSO_2PhC\mathit{H}_3$), 2.89 (1H, dd, *J*=13.8 and 7.9 Hz, $-COCHC\mathit{H}_2Ph$), 3.10 (1H, dd, *J*=13.8 and 6.9 Hz, $-COCHC\mathit{H}_2Ph$), 3.34 (3H, s, $-OC\mathit{H}_3$), 3.49-3.84 (12H, m, $-(O(C\mathit{H}_2)_2)_3O-$), 4.36 (1H, dd, *J*=7.9 and 7.3 Hz, $-COC\mathit{H}CH_2-$), 4.46 (2H, s, $-C{\equiv}CC\mathit{H}_2-$), 5.82 (1H, s, H-3), 7.08-7.63 (14H, m, H-1, 2', 3', 4', 6', $-COCHCH_2\mathit{Ph}$ and $NHSO_2\mathit{Ph}CH_3$), 8.14 (1H, s, H-3''), 8.80 (1H, s, H_3-5''); HRMS (ESI-TOF, positive mode): calcd for $C_{41}H_{50}N_4NaO_8S_1$ $[M+Na]^+$ 781.3247, found 781.3240.

References

1. Todoroki, Y. *et al.* Structure-activity relationship of uniconazole, a potent inhibitor of ABA 8'-hydroxylase, with a focus on hydrophilic functional groups and conformation. *Bioorganic Med. Chem.* **16,** 3141–3152 (2008).
2. Okazaki, M. *et al.* Abscinazole-E1, a novel chemical tool for exploring the role of ABA 8'-hydroxylase CYP707A. *Bioorganic Med. Chem.* **19,** 406–413 (2011).
